# Use of Chebychev Polynomials in Thin Film Computations

**DOI:** 10.6028/jres.063A.024

**Published:** 1959-12-01

**Authors:** Klaus D. Mielenz

## Abstract

From Herpin’s expression for the *m*th power of a multilayer matrix, very simple closed formulas are derived for the matrices and optical constants of any multilayer with a periodic structure.

According to Epstein’s theorem, any symmetrical multilayer is equivalent to a fictitious monolayer. A simple expression for the equivalent index and thickness of this monolayer is deduced for the case of a periodic and symmetrical sequence of equally thick films.

As compared to any other method of numerical computation, the suggested formulation provides a considerable saving of time and work. In a numerical example, this saving amounts to about 80 percent.

## 1. Fundamental Relationships [1, 2]^1^

**Figure 1:**
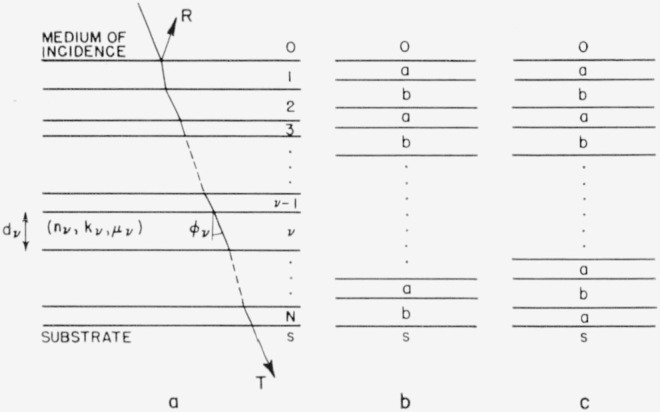
Optical multilayers (a) General case and denotations, (b) periodic, and (c) periodic-symmetrical multilayer. (In figures 1b and c, the individual layers a and b do not necessarily represent single films.)

The electromagnetic field at the plane of entry to a multilayer as in figure 1a is determined by

$$\begin{array}{l} \left({\;}_{H_{0}}^{E_{0}}\right)=A_{1}A_{2}\ldots A_{N}\left({\;}_{M_{s}}^{1}\right),\\ \;A_{\nu }=\left(\begin{array}{c} \cos\;B_{\nu }\;\frac{i}{M_{\nu }}\sin\;B_{\nu }\\ iM_{\nu }\sin\;B_{\nu }\;\;\cos\;B_{\nu } \end{array}\right),\left|A_{\nu }\right|=1, \end{array}$$ (1)

where

$$M_{\nu }=\frac{m_{\nu }}{\mu_{\nu }}=\frac{n_{\nu }(1-ik_{\nu })}{\mu_{\nu }},\;B_{\nu }=\frac{2\pi }{\lambda }m_{\nu }d_{\nu }\;\cos\;\phi_{\nu }$$ (2)

are the complex index and the optical thickness in phase units of the *ν*th layer (*n_ν_*=refractive index, *C_ν_* = absorption coefficient, *μ_ν_*=permeability, *d_ν_* = physical thickness, λ = wavelength, and *ϕ_ν_* = angle of incidence).

The amplitude transmission and reflection coefficients of the multilayer are

$$T=\frac{2M_{0}\sqrt{M_{s}+M_{s}^{*}}}{\sqrt{M_{0}+M_{0}^{*}}C},\;R=\frac{D}{C}$$ (3)

with

$$C=M_{0}E_{0}+H_{0},\;D=M_{0}E_{0}-H_{0}.$$ (4)

Various methods for computing *R* and *T* have been suggested in the literature. They all have the disadvantage of being based upon recurrence relations that make it necessary to calculate the desired quantities in a cumbersome stepwise manner. Although such a procedure seems to be inevitable in general, a much simpler approach is possible with “periodic” and “periodic-symmetrical” multilayers as represented in figures 1b and c. Since such multilayers are of considerable importance in thin film work, the computation method developed hereafter is of great practical significance.

## 2. “Periodic” Multilayers

A multilayer in which the same sequence of films is repeated twice or more often is a “periodic” multilayer. According to Herpin’s theorem [3] any multilayer, and therefore the fundamental period of layers as well, may be expressed as a fictitious bilayer the matrix of which we shall call $A_{a}A_{b}$. If the period occurs *m* times the matrix eq (1) reads

$$\left({\;}_{H_{0}}^{E_{0}}\right)={\left(A_{a}A_{b}\right)}^{m}\left({\;}_{M_{s}}^{1}\right).$$ (5)

## 3. “Periodic-Symmetrical” Multilayers

A multilayer for which the indices and thicknesses are the same as encountered from either side,

$$\left({\;}_{H_{0}}^{E_{0}}\right)=A_{\alpha }A_{\alpha -1}\ldots A_{2}A_{1}A_{2}\ldots A_{\alpha -1}A_{\alpha }\left({\;}_{M_{s}}^{1}\right),$$

is a “symmetrical” multilayer. It can be replaced by a fictitious monolayer (Epstein [4]).

If such a multilayer consists of [*m* + (1/2)] times a fundamental period,

$$\left({\;}_{H_{0}}^{E_{0}}\right)={\left(A_{a}A_{b}\right)}^{m}A_{a}\left({\;}_{M_{s}}^{1}\right),$$ (6)

it is a “periodic-symmetrical” multilayer.

## 4. The *m*th Power of a Matrix of Unity Determinant

A simple closed expression for ${\left(A_{a}A_{b}\right)}^{m}$ is the key to eqs (5) and (6). Herpin [5] has shown that the powers of a four-element matrix can be expressed by Lucas polynomials, and Abelès [6, 1] has observed that these are reduced to Chebychev polynomials if the basic matrix is of unity determinant (which is the case for multilayer matrices). This principle, however, has not been developed further since.

Let A be any four element matrix of determinant unity and write

$$A=\left({\;}_{a_{21}\;a_{22}}^{a_{11}\;a_{12}}\right)=a\sigma_{0}+b\sigma_{1}+c\sigma_{2}+d\sigma_{3},\;a^{2}-(b^{2}+c^{2}+d^{2})=1,$$ (7)

with

$$\sigma_{0}=\left(\begin{array}{cc} 1 & 0\\ 0 & 1 \end{array}\right),\;\sigma_{1}=\left(\begin{array}{cc} 0 & 1\\ 1 & 0 \end{array}\right),\;\sigma_{2}=\left(\begin{array}{cc} 0 & -i\\ i & 0 \end{array}\right),\;\sigma_{3}=\left(\begin{array}{cc} 1 & 0\\ 0 & -1 \end{array}\right)$$ (8)

(Pauli spin matrices). Then, we obtain

$$A^{2}=2aA-\sigma_{0}.$$ (9)

Following Herpin, we set

$$A^{m}=S_{m-1}A-S_{m-2}\sigma_{0},$$ (10)

whence we arrive at

$$A^{m+1}=S_{m}A-S_{m-1}\sigma_{0}=A^{m}A=S_{m-1}A^{2}-S_{m-2}A=2aS_{m-1}A-S_{m-1}\sigma_{0}-S_{m-2}A.$$

Putting

$$2a=a_{11}+a_{22}=X,$$ (11)

we find by comparison of coefficients, as recurrence formula for the *S*_m_’s,

$$S_{m}(X)=XS_{m-1}(X)-S_{m-2}(X).$$ (12)

Equation (9) yields the initial values,

$$S_{0}(X)=1,\;S_{1}(X)=X,$$ (13)

and then (12) leads to

$$S_{m}(X)=\sum_{\mu =0}^{\left[m/2\right]}{\left(-1\right)}^{\mu }\left({\;}_{\mu }^{m}\right)X^{m-2\mu },$$ (14)

where [m/2] denotes the largest integer contained in m/2, e.g., [5/2] = 2. Explicitly, we have

$$\begin{array}{l} S_{0}=1,\\ S_{1}=X,\\ S_{2}=X^{2}-1,\\ S_{3}=X^{3}-2X,\\ S_{4}=X^{4}-3X^{2}+1,\\ S_{5}=X^{5}-4X^{3}+3X,\\ S_{6}=X^{6}-5X^{4}+6X^{2}-1,\\ S_{7}=X^{7}-6X^{5}+10X^{3}-4X,\\ S_{8}=X^{8}-7X^{6}+15X^{4}-10X^{2}+1,\\ \;\text{etc}. \end{array}\}$$ (14a)

The *S_m_*’s defined by these equations are the Chebychev polynomials of the second kind [7],

$$S_{m}(X)=\frac{\sin\;(m+1)\theta }{\sin\;\theta },\;X=2\;\cos\;\theta ,$$ (15a)

or

$$S_{m}(X)=\frac{\sinh\;(m+1)\Phi }{\sinh\Phi },\;X=2\;\cosh\;\Phi .$$ (15b)

Another form is

$$S_{m}(X)=\frac{1}{2^{m+1}\sqrt{X^{2}-4}}[{\left(X+\sqrt{X^{2}-4}\right)}^{m+1}-{\left(X-\sqrt{X^{2}-4}\right)}^{m+1}].$$ (16)

For real arguments, *X=x*, these polynomials are also real (even though this is not obvious in eq (16) for |*x*|<2). If, in eqs (15), θ and Φ are to be real for reasons of convenience, (15a) must be used for |*x*|≤2, and (15b) for |*x*|≥2.

The desired matrix ${\left(A_{a}A_{b}\right)}^{m}$ may now be obtained as follows: Form

$$A_{a}A_{b}=\left(\begin{array}{cc} a_{11} & a_{12}\\ a_{21} & a_{22} \end{array}\right)\;\text{and}\;X=a_{11}+a_{22}.$$ (17a)

Then, find *S_m_*_−1_(*X*) and *S_m_*_−2_(*X*) and write

$${\left(A_{a}A_{b}\right)}^{m}=\left(\begin{array}{c} S_{m-1}a_{11}-S_{m-2}\;S_{m-1}\;a_{12}\\ \;S_{m-2}\;a_{21}S_{m-1}a_{22}-S_{m-2} \end{array}\right).$$ (17b)

## 5. Application to Multilayers

According to (5), (6), and (17), we have

$$F_{P}=S_{m-1}(X)\cdot \;F_{ab}-S_{m-2}(X)\cdot \;F_{s},$$ (18)

and

$$F_{PS}=S_{m-1}(X)\cdot F_{aba}-S_{m-2}(X)\cdot \;F_{a},$$ (19)

where *F* stands for either field strength, *E* or *H*, and because of (4) for the quantities *C* and *D* as well. The subscripts *P* and *PS* refer to the whole periodic and periodic-symmetrical multilayers, respectively; the subscripts *s, a*, *ab*, and *aba* refer to the uncoated substrate, the bottom mono-, bi-, and trilayers, respectively.

Thus, any of these quantities can be expressed as a simple linear combination of the corresponding quantities of much simpler multilayers.

By forming the matrix product in (16), one finds the argument of the Chebychev polynomials in (18) and (19),

$$X=2\;\cos\;B_{a}\;\cos\;B_{b}-\frac{M_{a}^{2}+M_{b}^{2}}{M_{a}M_{b}}\sin\;B_{a}\;\sin\;B_{b}.$$ (20)

## 6. Layers of Equal Thickness

The equations of section 5 are still further simplified if each film in the multilayer has the same optical thickness,

$$B_{a}=B_{b}=B.$$ (21)

Then, we have

$$X=2-\frac{{\left(M_{a}+M_{b}\right)}^{2}}{M_{a}M_{b}}\sin^{2}\;B.$$ (22)

While for the periodic multilayer the mathematical formulation itself is not simplified, a very simple formulation is obtained for the periodic-symmetrical multilayer:

With (21) and (22), the matrix of the trilayer (aba) can be shown to be

$$A_{a}A_{b}A_{a}=XA_{a}-A_{b}^{-1},$$ (23)

where

$$A_{b}^{-1}=(\begin{array}{c} \cos\;B\;-\frac{i}{M_{b}}\sin\;B\\ -iM_{b}\;\sin\;B\;\cos\;B \end{array})$$ (24)

is the inverse of Then, (17) and (12) show that

$${\left(A_{a}A_{b}\right)}^{m}\;A_{a}=S_{m}(X)A_{a}-S_{m-1}(X)A_{b}^{-1}.$$ (25)

Because of (13), (23) is contained in (25) as the particular case *m*=1.

Thus we see that, if all films are equally thick, it is not necessary to express the periodic-symmetrical multilayer in terms of the bottom trilayer and the uncoated substrate, as in (19). Instead, it can very simply be expressed in terms of the two basic matrices $A_{a}$ and $A_{b}$, without any need for multiplication of matrices whatsoever.

Epstein’s theorem [4], according to which any symmetrical multilayer is equivalent to a fictitious monolayer, was already mentioned. The index *M_m_* and the thickness *B_m_* of the monolayer corresponding to a periodic-symmetrical multilayer with equally thick films may now be obtained as follows:

Consider (25) and write

$${\left(A_{a}A_{b}\right)}^{m}A_{a}=\left(\begin{array}{c} \cos\;B_{m}\;\frac{i}{M_{m}}\;\sin\;B_{m}\\ iM_{m}\;\sin\;B_{m}\;\cos\;B_{m} \end{array}\right).$$ (26)

Then, comparison of coefficients yields, for the thickness *B_m_* and the index *M_m_* of the fictitious monolayer,

$$\cos\;B_{m}=[S_{m}(X)-S_{m-1}(X)]\;\cos\;B,$$ (27)

$$M_{m}^{2}=\frac{M_{a}S_{m}(X)+M_{b}S_{m-1}(X)}{\frac{1}{M_{a}}\;S_{m}(X)+\frac{1}{M_{b}}\;S_{m-1}(X)}.$$ (28)

(According to Epstein [4], the signs of *B_m_* and *M_m_* have to be chosen such that *B_m_*→0 for λ*→*∞, and that always Re(*M_m_*)≥0.)

## 7. Dielectric Multilayers

The above formulation constitutes a considerable simplification of practical computations.

An automatic computer can provide for itself the needed Chebychev polynomials by computing them according to eqs (14) or (16), regardless of whether *X* is a real or a complex number.

For desk calculations, however, numerical values of Chebychev polynomials of complex arguments cannot be found except with rather complicated calculations. This leads us to the restriction that *X* always should be a real number which will be true only if the multilayers are purely dielectric, and if the individual matrices $A_{a}$ and $A_{b}$ represent individual films. (The replacement of multilayers, even if purely dielectric, by fictitious mono- or bilayers may yield complex indices.) These assumptions lead to the important class of alternating dielectric layers, for which we have

$$M_{a}=m_{a}=n_{a},\;M_{b}=m_{b}=n_{b},$$ (29)

and (for equally thick layers that are quarter wave films at a wavelength λ_0_)

$$B_{a}=B_{b}=\beta =\frac{\pi }{2}\frac{\lambda_{0}}{\lambda }$$ (30)

so that

$$X=x=2-\frac{{\left(n_{a}+n_{b}\right)}^{2}}{n_{a}n_{b}}\;\sin^{2}\beta$$ (31)

is a real number.

For such real arguments, the Chebychev polynomials may be found with the aid of:

- *Numerical tables:* 12-decimal values of the first 12 *S_m_*′s, for 0≤*x*≤2 with intervals 0.001 in *x*, have been published by the National Bureau of Standards [7]. Jones and co-authors [8] have published similar but less complete tables. For negative arguments, find S*_m_*(|*x*|) and use the relation
  $$S_{m}(-x)={\left(-1\right)}^{m}S_{m}(x).$$ (32)
- Equation (15) *and tables of trigonometric or hyperbolic junctions:* If, for instance, *S*_4_(3.745) is looked for, one finds from the table that 3.745 = 2 cosh 1.24. Hence Φ=1.24, 5Φ = 6.20, and
  $$S_{4}(3.745)=\frac{\sinh\;6.20}{\sinh\;1.24}=\frac{246.37}{1.58311}=155.62.$$
- *Direct computation:* The *S_m_*′s may also be computed from eq (14) or (16). For small values of *m* it is easier to use eqs (14), but for large *m*′s (which, however, rarely occur in multilayer work) eq (16) is faster.

## 8. A Practical Example

Consider a high reflection multilayer consisting of 11 alternating zinc sulfide and magnesium fluoride films on glass,

$$\begin{array}{cc} n_{0}=1\;(\text{air}), & n_{a}=2.3\;(\text{ZnS}),\\ n_{b}=1.38\;({\text{MgF}}_{2}), & n_{s}=1.52\;(\text{glass}). \end{array}$$

Let all films be a quarter wave thick at λ_0_=5460.74 A, and compute the amplitude reflection coefficient *R* for λ=4358.35 A, i.e., for

$$\beta =\frac{\pi }{2}\frac{5460.74}{4358.35}=112{}^{\circ}\;45.86\prime ,\;\sin\;\beta =0.92211,\;\cos\;\beta =-0.38694.$$

Then we have according to (1), (24), and (25),

$$\left({\;}_{H_{0}}^{E_{0}}\right)=\left[S_{5}(x)\left(\begin{array}{cc} -0.38694 & \;0.40092i\\ \;2.12085i & -0.38694 \end{array}\right)-S_{4}(x)\left(\begin{array}{cc} -0.38694 & -0.66820i\\ -1.27252i & -0.38694 \end{array}\right)\right]\left(\begin{array}{c} 1\;\\ 1.52 \end{array}\right).$$

From (31), we obtain *x*= −1.62789, so that we may look up in reference [7]: *S*_5_(*x*) = 0.94004, *S*_4_*±*(*x*) = 0.07257.

Thence,

$$\begin{array}{l} \left({\;}_{H_{0}}^{E_{0}}\right)=\left[\left(\begin{array}{cc} -0.36374 & \;0.37688i\\ \;1.99363i & -0.36374 \end{array}\right)+\left(\begin{array}{cc} 0.02808 & 0.04849i\\ 0.09235i & 0.02808 \end{array}\right)\right]\left(\begin{array}{l} 1\\ 1.52 \end{array}\right)\\ \;=\left(\begin{array}{cc} -0.33566 & \;0.42537i\\ \;2.08603i & -0.33566 \end{array}\right)\left(\begin{array}{l} 1\\ 1.52 \end{array}\right)\\ \;=\left(\begin{array}{c} -0.33566+0.64656i\\ -0.51020+2.08603i \end{array}\right). \end{array}$$

Finally, (3) and (4) yield

$$R=\frac{\;0.17454-1.43917i}{-0.84586+2.73259i}.$$

Starting with the given *n*’s and with *β*, this result was obtained with 17 individual steps of multiplication or division and 9 steps of addition or subtraction. Four numerical values had to be looked up in tables.

In comparison hereto, it takes 96 multiplications, and 48 additions or subtractions, with 1 numerical value to be looked up, to arrive at the same result by means of the widely used recurrence method for admittances [9]. This may be estimated to be about five times as much work.

## Footnotes

1 Figures in brackets indicate the literature references at the end of this paper.
